# Single Cell Genomics: Advances and Future Perspectives

**DOI:** 10.1371/journal.pgen.1004126

**Published:** 2014-01-30

**Authors:** Iain C. Macaulay, Thierry Voet

**Affiliations:** 1 Single Cell Genomics Centre, Wellcome Trust Sanger Institute, Hinxton, Cambridge, United Kingdom; 2 Laboratory of Reproductive Genomics, Department of Human Genetics, KU Leuven, Belgium; University of Washington, United States of America

## Abstract

Advances in whole-genome and whole-transcriptome amplification have permitted the sequencing of the minute amounts of DNA and RNA present in a single cell, offering a window into the extent and nature of genomic and transcriptomic heterogeneity which occurs in both normal development and disease. Single-cell approaches stand poised to revolutionise our capacity to understand the scale of genomic, epigenomic, and transcriptomic diversity that occurs during the lifetime of an individual organism. Here, we review the major technological and biological breakthroughs achieved, describe the remaining challenges to overcome, and provide a glimpse into the promise of recent and future developments.

## Introduction

The cell is a fundamental unit of biology, in which the blueprint of the genome is transcribed and translated into biological form and function. Almost all of our current understanding of the genome and its regulation has been derived from studies carried out at the population level—typically thousands or millions of cells analysed in bulk. The resulting analysis, although unquestionably informative, often neglects any heterogeneity that occurs *within* the population of cells.

The genome, despite being widely thought of as stable throughout normal development, has a small probability of acquiring genetic mutations with every cell division [1], [2]. Over sufficient divisions, genomic heterogeneity within the organism—known as somatic variation—is a certainty. While such variation lies at the root of many disorders [3], [4], including cancer [5], recent studies revealed unexpected levels of genomic variation in normal and diseased tissue, suggesting higher rates of genetic lesion than previously expected [6]–[12]. Still, little is known about the rate and nature of DNA mutation and how this is influenced by genetic background, lifestyle, and many other factors.

The transcriptome is naturally more dynamic than the genome, reflecting the function—or type—of the cell. There is considerable evidence indicating that cell-to-cell variability in gene expression is ubiquitous, even within a phenotypically “homogeneous” population of cells [13]. The extent of transcriptional heterogeneity and the diversity of cell types in tissues remain, however, largely unknown.

The genomic and transcriptomic composition of individual cells is lost in conventional sequencing studies, which analyse DNA and/or RNA extracted from large populations of cells; and de novo genome mutation and transcriptomic variations in cells will be largely concealed in the bulk signal. Clear insights into many biological processes—from normal development to tumour evolution—will thus only be gained from a detailed understanding of genomic, epigenomic, and transcriptional variation at the single-cell level. Furthermore, some cell types are so rare that single-cell approaches become paramount to their identification and characterisation.

**Figure 1:**
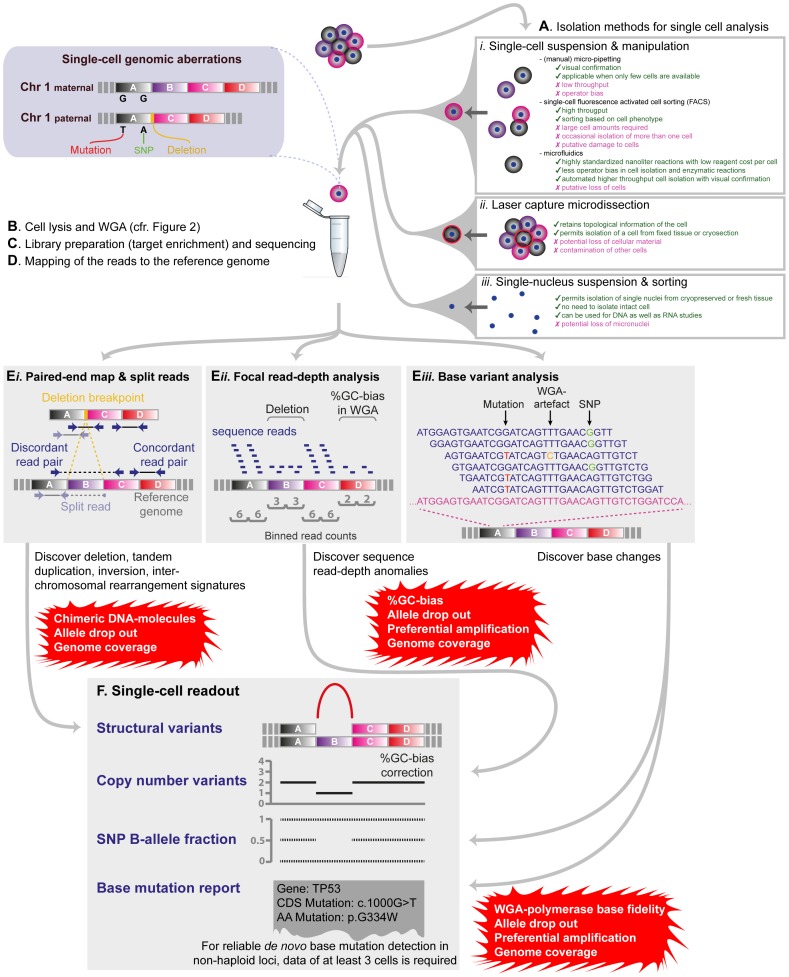
Detection of various classes of genetic variation using single-cell WGA-NGS approaches. A) The most prominent methods for (i–ii) isolating individual cells (including (i) creation of single-cell suspensions—usually by enzymatic tissue disaggregation—and subsequent cell isolation through manual micro-pipetting [37], [38], [57], [105], fluorescence-activated cell sorting [106], [107] or microfluidics devices [18], [81], [108], and (ii) laser capture microdissection [109], [110]) as well as (iii) isolating single nuclei [12], [32], [56], [111] are indicated, accompanied with particular advantages and disadvantages. A comprehensive review of single-cell isolation methods is presented by Shapiro et al. [112]. B–D) Subsequently, the cell is lysed and its genome amplified. A standard sequencing library can be prepared from the WGA product for paired-end (or single-end) sequencing. The resulting (short) sequence reads of the cell are mapped against a reference genome for variant discovery (E*i*–E*iii*). In all steps (E*i*–E*iii* towards F), various confounding factors resulting from the WGA process have to be considered in the analysis (indicated in red boxes). E*i*–F) Structural variants can be detected by analysing read-pairs which map discordantly to the reference genome, or by discovering split reads crossing a rearrangement. However, WGA can create various chimeric DNA molecules that resemble structural variants following paired-end sequence analysis of the WGA-product. E*ii*–F) Copy number variants are called by “binning” reads that map to particular regions of the genome. By comparing the read count per bin to the counts obtained in a reference sample [17], or an average read count per bin [32], a copy number profile can be calculated. However, single-cell copy number profiles can be distorted by ADO, PA, and %GC-bias during the WGA process. E*iii*–F) Single nucleotide variants (SNVs) can be detected in sequenced single-cell WGA products by aligning the reads with a reference genome. However, three cells carrying the same SNV are required to confidently call the variant.

Advances in techniques for the isolation of single cells (Figure 1A), whole genome or transcriptome amplification, and genome-wide analysis platforms—primarily next-generation sequencing (NGS) devices—paved the way for high-resolution analysis of the genome or transcriptome from one cell, which reveals previously obscured biological complexity.

## Single-Cell Whole-Genome Amplification: Methods and Limitations

**Figure 2:**
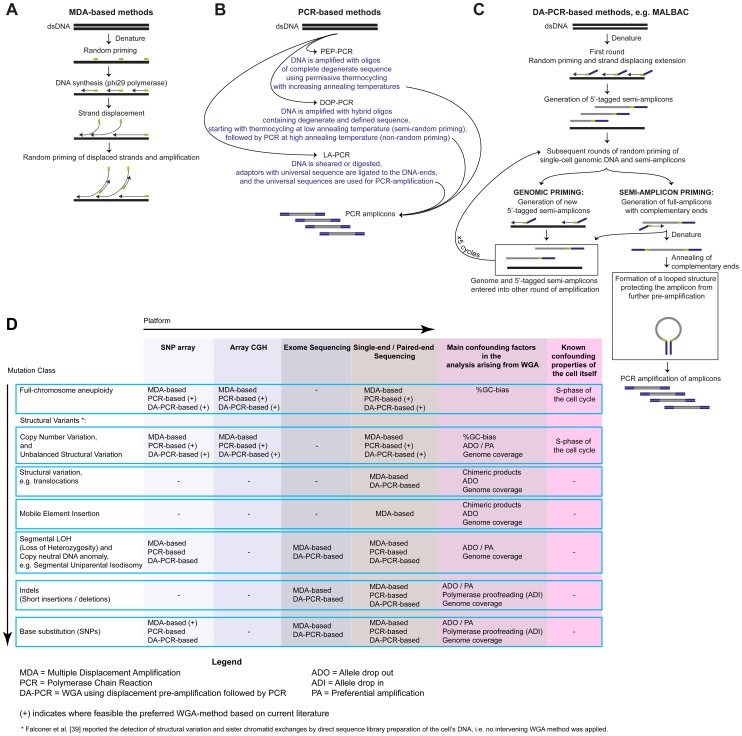
Overview of single-cell WGA approaches. A) Multiple displacement amplification (MDA) initiates with random priming of denatured single-cell DNA template, followed by a 30°C isothermal amplification using a DNA-polymerase with strand-displacement activity, typically phi29 [113]. When the 3′-end of a newly synthesized fragment reaches the 5′-end of an adjoining primed string of nucleotides, it will displace the latter, liberating single-stranded DNA for new primer annealing and DNA-synthesis. B) Primer extension pre-amplification (PEP)-PCR [114], degenerate-oligonucleotide primed (DOP)-PCR [115], and linker-adaptor (LA)-PCR [116] use PCR for WGA. C) WGA methods like PicoPlex [117], [118] and MALBAC [16] use displacement pre-amplification to generate PCR-amplifiable fragments (abbreviated as DA-PCR methods here). Specifically, MALBAC initiates with multiple rounds of displacement pre-amplification using a primer that anneals randomly throughout the genome, but contains a specific sequence allowing full amplicons to form looped pre-amplification products of a cell's template DNA. This looping protects previously copied segments from further pre-amplification. Multiple rounds of the displacement pre-amplification reaction, interspersed by a denaturation step, increase the probability that random priming will occur across the genome. D) Classes of genetic variation reported in single cells following WGA and analysis. The proofreading capacity of φ29 improves sequence fidelity during WGA [18], [37], [38], [119]. Furthermore, MDA amplifies the majority of a cell's genome and appears a preferred method for SNP genotyping [15], [17], [18] or base mutation detection [18], [37], [38], [120], but ADO and PA occurs. Following MDA, single-cell copy number profiles can be distorted [15], [17] —although improvements are emerging [19] —and chimeric DNA amplification products are created [17], [121]. In general, the (DA-)PCR-based WGA products more accurately preserve the copy number profile of the template genome [15]–[17] and can be used for SNP genotyping and base mutation detection [16].

A diploid human cell contains approximately 7 pg genomic DNA; necessitating amplification prior to microarray- or NGS-based analyses to detect various classes of genetic variation (Figure 1B–1F). Current whole-genome amplification (WGA) principles are based on Multiple Displacement Amplification (MDA), Polymerase Chain Reaction (PCR), or a combination of both (Figure 2A–2C).

Unfortunately, no WGA method is faultless, and their various imperfections can considerably affect the interpretation of the microarray or NGS readout [14]. The breadth of genomic coverage, the amplification bias due to local differences in richness for guanine and cytosine bases (%GC-bias), the prevalence of allelic drop outs (ADO), preferential allelic amplifications (PA), chimeric DNA-molecules, and nucleotide copy errors can vary significantly between different WGA approaches, making some methods better suited than others for detecting specific classes of genetic variation [14]–[17] (Figure 2D). A comparative analysis of all WGA methods, including the investigation of the advantageous effects of reducing the reaction volume to a nanoliter scale [18], [19], against a benchmark case is acute.

## Advances in NGS and Bioinformatics Permit High-Resolution Screening of a Single-Cell Genome

Single-cell WGA products have been analysed using a variety of high-throughput platforms, including DNA-microarrays, SNP-arrays, and NGS (Figure 2D). A key difficulty in the interpretation of single-cell WGA data on any platform is the separation of the numerous WGA artifacts from the genuine genetic variants present in the template genome.

Standard DNA-microarrays can detect copy number variations (CNVs) larger than 2.5 Mb from a single-cell genome [20]–[22], while targeted array comparative genomic hybridizations can discover approximately 1 Mb-sized DNA imbalances [23], although remarkably, CNVs as small as 56 kb in single-cell PCR-based WGA products have been detected [24]. Similarly, SNP-arrays can find copy number aberrations encompassing millions of bases in a cell [25]–[28], but have the advantage of enabling the discovery of copy neutral DNA anomalies and regions of loss-of-heterozygosity (LOH), and allow inferring genome-wide haplotypes [29]–[31].

NGS has a number of advantages over microarrays enabling improved resolution and accuracy in variant calling [14]. First, NGS can examine every nucleotide amplified from the cell and allows genome-wide discovery of the full spectrum of DNA mutations (Figure 1E), while microarrays only probe for certain CNV loci (Figure 2D). Secondly, sequencing provides digital precision, with one digital unit representing a mapped sequence read. Finally, paired-end sequencing and mapping discloses the linkage between both ends of each linear DNA-molecule in a sequencing library of a single-cell WGA product, allowing the identification of structural variations via read-pairs mapping discordantly to the reference genome (Figure 1E *i*).

Analytical challenges remain in interpreting single-cell NGS data for the full spectrum of genetic variants. Although WGA imperfections due to genome base composition (e.g. %GC-bias) can be computationally corrected for [17], [32], the potential for PA and ADO can still generate local distortion in copy number, requiring distinct analyses to distinguish genuine copy number changes from WGA artefacts. Allelic fractions of heterozygous SNPs [26], [33], [34] or aberrantly mapping read pairs following paired-end sequencing of the WGA product [17] can be used to increase confidence in CNV measurements (Figure 1B–1F). For instance, a real deletion of a diploid locus should show LOH and discordantly mapping read-pairs that explain the DNA loss. Furthermore, the cell cycle stage of the isolated cell must be considered, further complicating the analysis, as cells in S-phase demonstrate a dynamic copy number profile, leading to false structural DNA-imbalance discoveries [35].

The identification of the full spectrum of intra- and inter-chromosomal (un)balanced structural variants in a single-cell WGA product is still in its infancy—the main difficulty being to filter true structural variants from chimeric DNA generated during WGA, as well as issues with genome coverage (Figure 1E *i*, 1F). Although filters have been designed to permit the detection of the structural architecture of DNA copy number variation [17] and even to detect L1-retrotransposition [36], many structural variants are still missed in single-cell analyses. Base alterations, such as SNPs, can be detected in single-cell WGA products (Figure 1E *iii*). However, to call accurate and reliable base substitutions in non-haploid loci, one requires the data of at least three cells to discriminate the variant from a WGA or sequencing error [16], [37], [38], and as such, detailed characterisation of extremely rare cells or sub-clones within populations may not be possible. Despite these hurdles, several groups have proven the efficacy of single-cell NGS to detect multiple classes of mutation within a genome and even to detect sister chromatid exchanges following single-cell Strand-seq [39]. Step-by-step bioinformatics protocols for analyzing Strand-seq data [40] as well as for copy number profiling single cells through NGS [32] or microarray analysis [34] and commercial solutions (e.g. platforms used within [21], [41]) are surfacing.

## Single-Cell Genomics Reveals the Extent of Somatic Variation in Development and Disease

The study of multiple classes of mutations at the single-cell level revealed startling insights into the genomic variation that can occur during the human life cycle. Following single-cell genome-wide analysis, up to 7% [18], [42] and up to 70% [43]–[45] of male and female gametes, respectively, contain numerical chromosomal anomalies due to meiotic mis-segregations. Furthermore, sequencing of haploid single sperm cells revealed a base mutation rate of 2–4×10^−8^—which is severalfold higher than measurements from genome-sequenced pedigree data [46]. Single-cell analyses of human embryos following in vitro fertilization (IVF) demonstrated that the very first cell cycles of human life are prone to numerical and structural chromosome instability [17], [25]–[27], [44], [47]–[51]. Various observations indirectly hint that an in vivo conceptus faces a similar period of increased genomic vulnerability [52]–[55], suggesting that the first cell divisions may represent a spring of DNA mutation, which does not necessarily undermine normal development [8], but can lead to a spectrum of conditions, including loss of conception, genetic disorders, and genetic variation development.

Several studies sequenced and dissected cancer genomes to single-cell resolution, with the aim of understanding tumour development and progression of the disease. Copy number landscapes of single nuclei from primary mammary ductal carcinomas and a paired metastatic liver tumour were generated following low-coverage sequencing. This revealed various chromosomal rearrangements, followed by distinct phases of clonal expansion during tumour evolution and metastasis [56]. Subsequent single-cell exome sequencing studies in bladder [57], kidney [38], and hematopoietic neoplasms [37] provided a detailed characterisation of base mutations in specific genes. Similarly, whole-genome sequencing of multiple MALBAC-amplified cells (Figure 2C) revealed a base mutation rate of a cancer cell line to be increased 10-fold when compared to estimated germ-line ciphers [16]. Furthermore, by sequencing daughter cells of a single mitotic division, the acquisition of new CNVs could be demonstrated for a breast cancer cell line [17].

Single-cell genome sequencing continues to provide new insights into genomic (in)stability in various cell types and developmental processes [12]. It will lead to a better understanding of the acquisition of genetic changes during induced pluripotent stem cell derivation and reprogramming, and to insights in the effects of mutagens, carcinogens, ageing, or germ-line genetic profile on general mutation burden. The methods will enable dissection of the genetic content of individual cells in normal organs, premalignant states, and established tumours, providing insights into the operation of genome maintenance in health and disease.

## Single-Cell Genomics in the Clinic

Single-cell genomics is providing cutting-edge clinical applications, notably in the genetic diagnosis of preimplantation human embryos following IVF (reviewed by Van der Aa et al. [58]). Furthermore, developing embryos shed cells in the maternal blood stream following implantation; the potential to capture and analyse such circulating foetal cells [59], [60] may broaden the scope and precision of current non-invasive prenatal testing of circulating foetal DNA in maternal plasma. Single-cell genomics is also applied for studying blood-borne circulating tumour cells (CTCs) [61]–[63], derived from a solid tumour, to investigate the value of CTCs, in addition to tumour-cell–free DNA [64], for guiding diagnosis, prognosis, and treatment of the cancer.

## Single-Cell Whole-Transcriptome Amplification: Methods and Limitations

**Figure 3:**
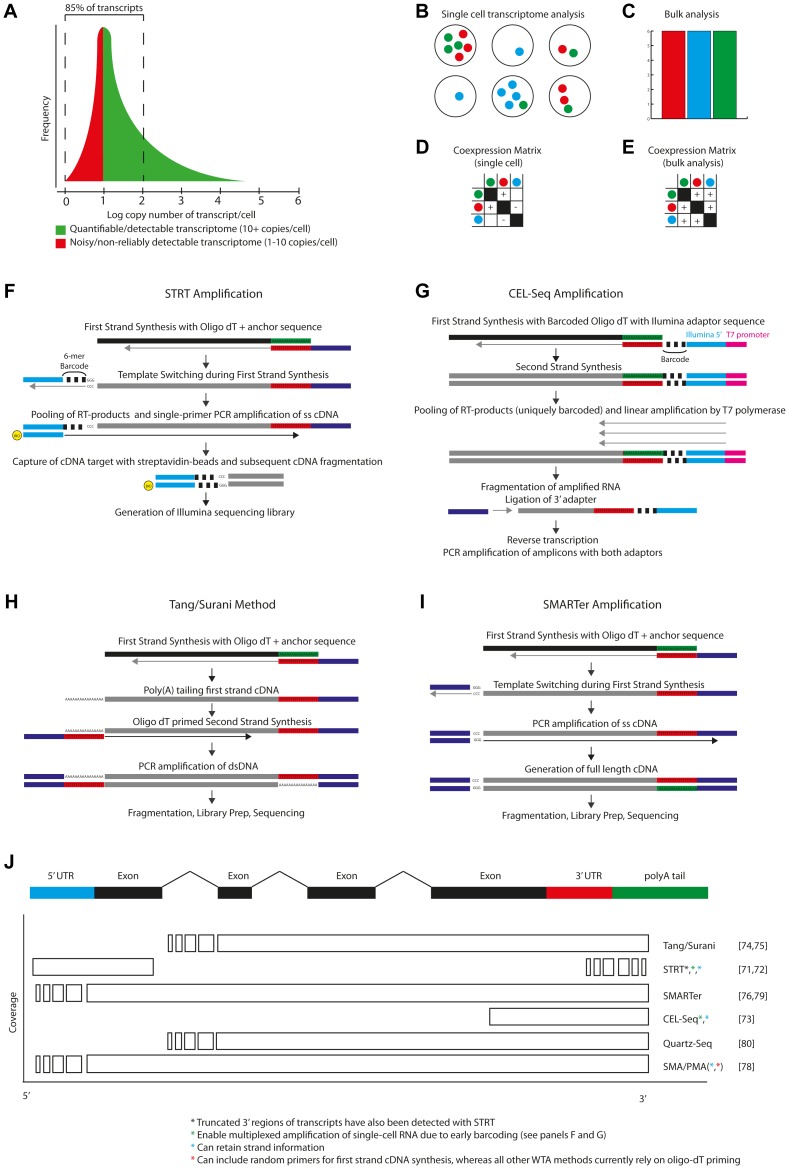
Single-cell transcriptomics. A) A single cell is thought to contain a few hundred thousand mRNA transcripts, present in a log-normal distribution of abundances, with as much as 85% speculated to be present between 1–100 copies. Current amplification methods are thought to reliably detect transcripts at >5–10 copies per cell. B) Single-cell transcriptomes reveal heterogeneity in gene (co)expression that bulk analysis would not permit. Six single cells are shown, with heterogeneous expression of three genes. C) Bulk analysis would detect uniform expression of all three genes. D–E) Single-cell analysis would reveal underlying heterogeneity but also indicate that two of these genes showed a pattern of co-expression. F) STRT-Seq [71], [72] is initiated by reverse transcription using an oligo-dT adaptor-primer. At the 5′ cap of the transcript, non-template nucleotides are added by the reverse transcriptase, permitting hybridisation of a barcoded template-switching adaptor-primer. Following pooling of barcoded RT-products, PCR amplification is performed, after which the 5′-ends are captured and sequenced. G) CEL-Seq [73] is initiated using a barcoded oligo-dT primer, which also contains a 5′ adaptor sequence and T7 RNA-polymerase priming site. Complimentary RNA is generated from the cDNA by T7 RNA polymerase. The cRNA is then fragmented and prepared for (3′-end) paired-end sequencing. H) The Tang/Surani method [74], [75], and improved derivatives [69], first generates, then 3′ polyadenylates, first strand cDNA. Priming with adaptor-conjugated oligo-dT generates double stranded cDNA, which is then amplified by PCR and sequenced. I) The SMARTer method [76], [79] uses template-switching to generate a full-length transcript with adaptor sequences at both ends. These sequences are then used to prime PCR amplification of the transcriptome. The full-length cDNA is used as input for sequencing. J) Overview of the sequence coverage of a typical transcript which would be obtained by each of the currently available single-cell RNA-seq methods [71]–[80].

A human cell likely contains less than 1 pg of mRNA [65]. Transcripts are thought to be expressed over several orders of magnitude, where many transcripts have low level expression (5–20 transcript copies per cell) [66], with more than 85% having less than 100 copies per cell (Figure 3A). The transcriptome of an individual cell is not fixed, but reflects the functionality of the cell, as well as its responses to acute extrinsic and intrinsic stimuli. In addition to this “regulated” heterogeneity, there is also transcriptional “noise”—heterogeneity which emerges from the kinetics of transcription and mRNA decay between cells within a population. In gene expression analyses of bulk cell populations, it may be impossible to distinguish changes in expression from changes in the cellular composition of the population. Similarly, genes perceived to be co-expressed at the population level may in fact be mutually exclusively expressed when observed at the single-cell level (Figure 3B–3E).

Single-cell reverse transcription and whole-transcriptome amplification (WTA) methods have been developed to permit qPCR [67]–[69], microarray [70], and more recently, NGS analyses of the transcriptomes of single cells. Various single-cell RNA-seq methods now exist, each offering an overview of either the 5′end [71], [72], 3′end [73] or even the full length [74]–[80] of transcripts from a single cell (Figure 3F–3J).

Reverse transcription, the initial step in each RNA amplification method, and subsequent conversion of cDNA into amplifiable molecules are likely key limiting factors in the detection and quantification of transcripts in single cells. It is estimated that on average only 5–25% of mRNA-molecules are converted to amplifiable cDNA [72]. Additionally, PCR-based amplification methods have the potential for non-linear amplification, resulting in the distortion of the relative abundance of transcripts. In vitro transcription based WTA-methods, such as CEL-Seq [73] (Figure 3G), may arguably avoid such complications through linear amplification of the transcriptome. Furthermore, nanoliter-scale reactions can demonstrate benefits over microliter-scale processes [81].

## NGS and Bioinformatics Analyses of Single-Cell Transcriptomes

At the most basic level of analysis, a single-cell RNA-seq experiment gives a readout of the abundance of a transcript within a cell. For 3′- or 5′-end sequencing, this is calculated simply as the number of reads mapping to a particular transcript, normalised to the overall number of reads for that cell. If full length RNA is analysed, the number of reads mapping to each transcript is normalised both for the number of reads per cell and, additionally, for transcript length.

Comparative analyses can be applied to measure differences in normalised gene expression between cells. Genes with heterogeneous expression can be identified by their variability within the population; subsequent clustering of variable genes may allow identification of subsets of genes that are co-expressed within a sub-population of cells. Such approaches have been used to dissect specific “bimodal” gene expression patterns within a population of cells [82] and to distinguish co-expression modules in early embryogenesis [83]. While many of the analytical tools for “bulk” mRNA sequencing are also applied for single-cell data, necessary tools specific for single-cell transcriptomics are starting to emerge [72], [84], [85].

The broad range of transcript abundance in a single cell presents a particular challenge for any amplification method—transcripts present at extremely low levels can still have important biological consequences, and yet, they may be undetectable due to inefficiency of the amplification approach. Even if they are detectable, the influence of technical noise and stochastic effects at these low levels may result in unreliable measurements of relative abundance within or between individual cells. Thus, a major challenge in quantitative analysis of single-cell transcriptome data is understanding technical noise within or between the samples [86]. The inclusion of RNA spike-ins, such as those developed by the External RNA Controls Consortium (ERCC) [87], can give particular insights into the relative efficiency, detection limits, and technical noise of each amplification method [84], [85]. Furthermore, single molecule counting approaches—which incorporate a unique identifier into every molecule prior to amplification—will indicate the extent to which individual RNA molecules are amplified [88].

## Insights from Single-Cell Transcriptomics

Single-cell RNA-seq has already been applied to catalogue allele specific expression and expression of long non-coding RNAs in single blastomeres [74], [89] as well as to dissect transcriptional programmes in single cells derived from human and mouse embryos [83], [90], revealing insights into the transcriptional modules that are activated at critical points during development. SMARTer WTA [76], [79] (Figure 3I) has been used to detect differential exon usage between single cells [82] and to demonstrate a bimodality in gene expression in a phenotypically homogeneous population of bone marrow dendritic cells upon treatment with lipopolysaccharide (LPS) [82]. Here, even genes which were highly expressed were restricted to only a subset of the population—an observation that would have been missed had the population been analysed at a bulk level.

Single-cell transcriptomics has the power to dissect mixed populations of cells; conversely, if only limited material is available, it may permit characterisation of the transcriptome of extremely rare cells, such as CTCs [76].

## The Future: Less Amplification, More Cells, More Types of Data

Many undesirable consequences of WGA and WTA remain to be solved. The ability to reduce [19] or even eliminate amplification of DNA or RNA before sequencing could increase the accuracy and reliability of single-cell analysis. Input requirements for library preparation continue to reduce, and direct library preparation from single-cell genomes has been demonstrated [39], [91]. The capacity to directly sequence unamplified DNA and RNA derived from single cells, however, requires further innovation, though direct sequencing of single molecules is already a possibility for DNA and RNA [92], [93]. Furthermore, translation of molecular counting principles to single-cell DNA sequence analyses may allow more accurate measurements of CNVs and enable base-error correction [88], [94], in addition to haplotyping approaches. Interpreting the full epigenomic status of a cell remains a challenge, but protocols for single-cell DNA-methylation [95]–[98] and chromosome conformation capture [99] assays are emerging. Excitingly, methodology to analyse both the (epi)genome and transcriptome of the same cell in parallel is in development and will offer a powerful platform to analyse the exact relationship between genomic variation, regulation, and gene expression.

Typical single-cell sequencing studies have focussed on small numbers of cells (10 s–100 s) but have already demonstrated the potential to distinguish complex heterogeneity at this level. The application of automated cell capture, amplification, and library preparation systems—particularly those utilising nanofluidics approaches—will dramatically increase the scale and affordability of single-cell analyses, such that much larger experiments will emerge.

## Towards a Phylogenetic Tree of a Human Lifetime, and the Discovery of New Cell Types

Studies like the 1000 Genomes Project have contributed greatly to our understanding of genetic and phenotypic variation amongst individuals within a population. However, these studies are grounded on the assumption that the genome of the individual is “fixed” in tissues throughout life.

Considerable evidence is emerging that somatic genomic variation is both common and dynamic in a human being [12], [100]–[104], although little is known about its scale, origin, rate, and nature. Dedicated bioinformatics analyses can extract only the most prevalent heterogeneities (>5% of cells) from populations of cells, representing likely just the tip of the iceberg. To truly understand the full extent of genomic heterogeneity, from conception to death, single-cell genomes must be investigated.

Large-scale single-cell sequencing projects, performed on cells from endodermal, mesodermal, and ectodermal tissues from an individual, will enable construction of a phylogenetic tree of a human lifetime and mapping of the contribution of genetic heterogeneity to the organism. Concurrent single-cell (epi)genomic/transcriptomic studies, on a large enough scale, will allow definitive sub-classification of cell types by gene expression profiles and (epi)genetic status, replacing or enhancing the current schema. Such studies will reveal, in ways that studies of bulk populations cannot address, the relationship between genome sequence, epigenetic status, and gene expression, determining the functional capacity of the cell.

## Conclusion

The last few years have seen rapid development of technologies and methods that permit highly detailed analysis of the genome and transcriptome of a single cell. In parallel, various observations have been made that suggest that both genomic and transcriptomic heterogeneity within an organism may have been considerably underestimated. Single-cell approaches now stand poised to illuminate this new layer of biological complexity during normal development and disease.
